# Intensive topical steroid regimen for enhanced very early recovery after small incision lenticule extraction

**DOI:** 10.1007/s10792-023-02827-7

**Published:** 2023-08-10

**Authors:** Qinghong Lin, Zhengwei Shen, Xingtao Zhou

**Affiliations:** 1 Eye Institute and Department of Ophthalmology, Eye and ENT Hospital, Fudan University, No.83 Fenyang Road, Shanghai, 200000 China; 2 Department of Refractive Surgery, Bright Eye Hospital, Fuzhou, 350000 China; 3 Department of Refractive Surgery, Wuhan Bright Eye Hospital, No.179 Zhongshan Road, Wuhan, 430000 China

**Keywords:** Myopia, Small incision lenticule extraction (SMILE), 0.1% Fluorometholone, Defined daily dose (DDD), Early phase

## Abstract

**Objective:**

This study aims to investigate the topical steroid regimen after small incision lenticule extraction (SMILE) for its effect on very early restoration of visual quality.

**Methods:**

A total of 180 patients (360 eyes) who underwent SMILE were enrolled. These patients were randomly assigned to three groups, with 60 patients in each group. The only difference among these three groups was the administration of 0.1% fluorometholone (FML) eye drops within two hours after SMILE: no FML in group A, 0.1% FML once every hour in group B and 0.1% FML once every half hour in group C. The corrected distance visual acuity (CDVA), objective scattering index (OSI), modulation transfer function (MTF) cut-off, Strehl ratio (SR) and incidence of subjective symptoms were evaluated preoperatively, at 2, 4 and 24 h and one week after SMILE.

**Results:**

The CDVA, MTF cut-off and SR values were significantly higher in group C, when compared to the other two groups, at 2 and 4 h after SMILE (*p* < 0.05). Furthermore, the OSI and incidence of subjective symptoms were significantly lower in group C, when compared to the other two groups, at 2 and 4 h after SMILE (*p* < 0.05). However, no significant differences in CDVA, MTF cut-off, SR, OSI and the incidence of subjective symptoms were detected among the three groups at 24 h and one week after SMILE (*p* > 0.05).

**Conclusion:**

The administration of 0.1% FML eye drops every half hour within two hours after SMILE accelerates the restoration of visual and optical quality, and reduces the incidence of subjective symptoms during the very early phase after surgery.

## Introduction

Small incision lenticule extraction (SMILE) and femtosecond laser-assisted in situ keratomileusis (FS-LASIK) are presently the two popular techniques for myopia treatment [1]. SMILE is an advanced technique based on FS-LASIK, with less injury to the corneal epithelium and nerves, and this has been proven to be safer, with fewer complications [1]. Both techniques have similar visual quality outcomes in the long term [2–4], and SMILE is expected to gradually replace FS-LASIK in refractive surgery clinics [5–7]. However, some patients continue to choose FS-LASIK, because SMILE has poorer visual function outcomes during the very early phase after surgery [8]. Thus, this issue needs to be addressed to promote SMILE as a more popular procedure for correcting myopia and myopia astigmatism.

The postoperative treatment regimens for SMILE and FS-LASIK are usually similar, which include the administration of antibiotics and steroid eye drops for four times a day after surgery [9–11]. Steroid eye drops can reduce the inflammatory and wound healing responses of the corneal tissue and accelerate visual recovery after surgery [10, 12, 13]. Therefore, the present study aimed to determine the efficacy of the intensive topical steroid regimen on clinical outcomes at the very early phase (within 24 h) after SMILE.

## Patients and methods

### Ethics statement

The present prospective study was approved by the Ethics Committee of Fujian Medical University (Fuzhou, China) and was conducted in compliance with the principles of the Declaration of Helsinki. A written informed consent was obtained from all patients.

### Participants and treatment procedures

In the present study, 180 patients (360 eyes), who underwent SMILE at the Eye Center of Fujian Medical University between July and November 2019, were randomly divided into three groups using a random number table, with 60 patients (120 eyes) in each group. The protocol for the SMILE procedure was the same as the protocol described by Liu et al. [8]. Briefly, SMILE was conducted using the VisuMax femtosecond laser (Zeiss, Oberkochen, Germany), with a 7.5-mm-diameter and 120-µm-thick cap and a 6.5-mm-diameter posterior lenticule surface. The corneal incision was 2 mm long (32°) and performed at the 11 o’clock position. In order to maintain investigator masking, the investigators were assigned into three groups: eye drops administration, examination and data analysis, respectively. The investigator assigned for each group was blinded to the information of the other groups, in order to avoid bias in the results.

All patients received 0.5% levofloxacin eye drops (Santen Pharmaceutical Co., Ltd., Osaka, Japan) for four times a day, which was immediately administered after the SMILE procedure, and 0.1% fluorometholone (FML) eye drops (Santen Pharmaceutical Co., Ltd., Osaka, Japan), which were administered within two hours after the SMILE procedure (except for group A), and for four times a day starting from the second day after the SMILE procedure. According to the regimen for FML administration after SMILE, the patients were divided into three groups: no FML in group A, 0.1% FML once every hour (twice in total) in group B and 0.1% FML once every half hour (four times in total) in group C.

### Examination and measurements

The corrected distance visual acuity (CDVA), mean spherical equivalent (MSE) and intraocular pressure (IOP) were measured before SMILE, at 2, 4 and 24 h, one week after SMILE, and at each follow-up visit thereafter. The CDVA was examined using a standard Landolt visual acuity chart, and the values were converted to the logarithm of the minimal angle resolution (logMAR) of visual acuity for the statistical analysis. The MSE was measured using an open-field autorefractor (Grand Seiko WR-5100 K; RyuSyo Industrial Co., Ltd., Kagawa, Japan), and the IOP was measured using a non-contact tonometer (TX-20; Canon, Tokyo, Japan).

The optical quality was measured with the Optical Quality Analysis System II (OQAS; Visiometrics, Terrassa, Spain) using the dual-channel technique [14]. The cut-off data for the modulation transfer function (MTF), Strehl ratio (SR) and objective scattering index (OSI) were collected preoperatively, at 2, 4 and 24 h and one week after SMILE for comparisons among the three groups.

The incidence of subjective symptoms after SMILE was collected from all patients, which included foreign body sensation, eye soreness, eye dryness and blurred vision, at 2, 4 and 24 h and one week after SMILE.

### Statistical analysis

SPSS 18.0 (SPSS Inc., Chicago, Illinois, USA) was used for the statistical analysis. The variables used for the present study comprised the parametric data, and these were tested as normal distribution. All measurements were repeated for three times, and the mean values were used for the analysis. Two-way ANOVA was used to compare the differences in measurements between groups. A *p* < 0.05 was considered statistically significant.

## Results

### Study participants

The demographics of the study participants are summarized in Table 1. There were no significant differences in CDVA, MSE and IOP. Furthermore, the distribution of age and gender was matched between groups. There were no intra-operative complications in any of the patients.

**Table 1:** Demographic information of patients at pre-operation

	logMAR	MSE (D)	IOP (mmHg)	Age	Gender	
					Male	Female
Group A	0.04 ± 0.08	− 6.23 ± 1.33	13.56 ± 2.45	21.56 ± 3.42	32	28
Group B	0.06 ± 0.06	− 5.98 ± 1.65	14.03 ± 2.21	20.88 ± 3.67	34	26
Group C	0.03 ± 0.10	− 6.05 ± 1.42	14.11 ± 1.98	22.01 ± 2.42	30	30
*F*-value	1.76	1.53	1.48	1.44		
*p*-value	> 0.05	> 0.05	> 0.05	> 0.05		

*p-value, comparisons among groups

### CDVA, MTF, SR and OSI after SMILE

**Fig. 1:**
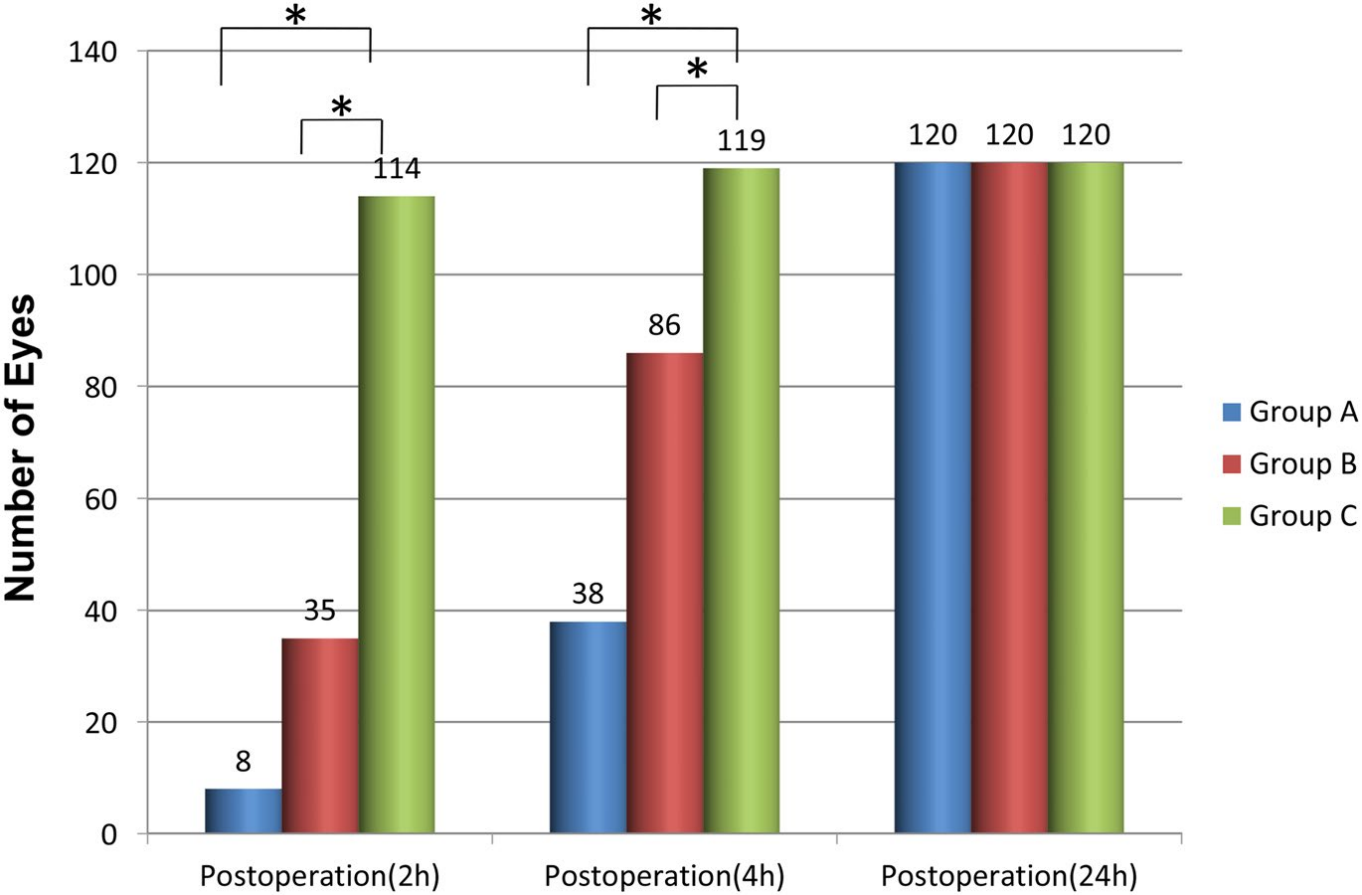
The number of eyes in each group that had a visual acuity of higher than 1.0 (logMAR, 0.00) at 2, 4 and 24 h after small incision lenticule extraction (SMILE), *p < 0.05

In groups A, B and C, in which different 0.1% FML regimens were administered, CDVA was examined at 2, 4 and 24 h and one week after SMILE. The number of eyes in each group, with a CDVA higher than 1.0 (logMAR, 0.00), is presented in Fig. 1. The number of patients in group C, who recovered to normal visual acuity at 2 and 4 h after surgery (Tables 2 and 3), was significantly higher, when compared to the number of patients in groups A and B (*p* < 0.05). However, at 24 h and one week after surgery (Tables 4 and 5), all eyes that underwent SMILE achieved normal visual acuity, and there were no differences between groups (*p* > 0.05).

**Table 2:** Measurements at postoperative two hours

	logMAR	MTF cut-off	SR	OSI
Group A	0.22 ± 0.14	11.53 ± 8.76	0.08 ± 0.10	3.63 ± 1.52
Group B	0.12 ± 0.10	20.70 ± 8.43	0.12 ± 0.05	2.83 ± 1.36
Group C	− 0.04 ± 0.08	30.88 ± 6.71	0.20 ± 0.06	1.26 ± 0.72
*p*-value	< 0.05	< 0.05	< 0.05	< 0.05

*p-value, comparisons among groups

**Table 3:** Measurements at postoperative four hours

	logMAR	MTF cut-off	SR	OSI
Group A	0.18 ± 0.16	18.62 ± 9.56	0.11 ± 0.12	2.53 ± 1.69
Group B	0.10 ± 0.13	28.60 ± 7.84	0.16 ± 0.10	2.01 ± 1.54
Group C	− 0.08 ± 0.09	37.98 ± 5.66	0.22 ± 0.09	0.77 ± 0.52
*p*-value	< 0.05	< 0.05	< 0.05	< 0.05

*p-value, comparisons among groups

**Table 4:** Measurements at postoperative 24 h

	logMAR	MTF cut-off	SR	OSI
Group A	− 0.10 ± 0.11	35.12 ± 6.33	0.20 ± 0.11	0.78 ± 0.34
Group B	− 0.11 ± 0.12	37.20 ± 7.74	0.22 ± 0.12	0.76 ± 0.51
Group C	− 0.13 ± 0.06	38.10 ± 5.98	0.21 ± 0.10	0.69 ± 0.62
*p*-value	> 0.05	> 0.05	> 0.05	> 0.05

*p-value, comparisons among groups

**Table 5:** Measurements at postoperative one week

	logMAR	MTF cut-off	SR	OSI
Group A	− 0.11 ± 0.10	37.10 ± 6.12	0.21 ± 0.10	0.66 ± 0.54
Group B	− 0.13 ± 0.13	36.22 ± 6.47	0.21 ± 0.12	0.64 ± 0.63
Group C	− 0.14 ± 0.08	37.23 ± 7.17	0.22 ± 0.09	0.63 ± 0.61
*p*-value	> 0.05	> 0.05	> 0.05	> 0.05

*p-value, comparisons among groups

The MTF cut-off, SR and OSI at 2, 4 and 24 h and one week after SMILE are presented in Tables 2, 3, 4 and 5, respectively. Significant differences in the MTF cut-off, SR and OSI were observed between groups at 2 and 4 h after surgery. Furthermore, higher MTF cut-off and SR values were observed in group C, when compared to groups A and B (*p* < 0.05), while lower OSI values were observed in group C, when compared to groups A and B (*p* < 0.05). However, there was no difference in MTF cut-off, SR and OSI values between groups at 24 h and one week after surgery (*p* > 0.05).

The incidence of subjective symptoms after SMILE was recorded (Table 6). From two hours to one week after SMILE, a reduction in complaints on subjective symptoms was observed for all patients. The comparisons between groups revealed significantly lower incidences of subjective symptoms at 2 and 4 h after surgery in group C (*p* < 0.05). However, there was no difference in the incidence of subjective symptoms between groups at 24 h and one week after surgery (*p* > 0.05).

**Table 6:** The incidence of subjective symptoms in the different groups (%)

	Post-operation (2 h)	Post-operation (4 h)	Post-operation (24 h)	Post-operation (1 week)	*P*-value
Group A	98.33%	68.33%	10.83%	5.00%	< 0.05
Group B	66.67%	35%	11.67%	5.00%	< 0.05
Group C	38.33%	18.33%	10.00%	3.33%	< 0.05
*p*-value	< 0.05	< 0.05	> 0.05	> 0.05	

*p-value, comparisons among groups

## Discussion

The early pathological changes induced by the SMILE or FS-LASIK procedure are inflammation, keratocyte apoptosis and proliferation [2]. In clinic, corneal edema and opacity may be observed at the early postoperative phase [8, 15, 16]. A postoperative topical steroid regimen, such as 0.1% FML eye drops for four times a day, is usually applied to attenuate these reactions and accelerate the restoration of visual quality [9–11, 17–19]. Using the routine postoperative regimen, SMILE and FS-LASIK are comparable, in terms of long-term visual quality [20]. However, the short-term visual outcome of FS-LASIK is better, when compared to that of SMILE, in the early postoperative phase, and this may be due to the interface haze after SMILE [8, 18, 21, 22]. Therefore, the present study investigated the intensive steroid regimen in the very early phase (within two hours) after SMILE, in order to determine whether this can accelerate the restoration of visual and optical quality.

Focus was given on the very early postoperative treatment, because clinical outcomes in the early phase after refractive surgery are significantly important for patients. The majority of the patients are young college students or workers. Thus, they expect that the laser-assisted refractive surgery would allow for rapid recovery, and enable them to return to work on the same day of the surgery. For this reason, some patients prefer FS-LASIK to correct their myopia or myopic astigmatism. SMILE was developed as an advanced technique, with fewer dry eye symptoms, no cap-related complications and more predictable visual correction [21–23]. This technique is an alternative to FS-LASIK and is more popular in clinic [5–7]. Thus, the present study aimed to improve the regimen and provide satisfactory early phase outcomes for patients who choose SMILE.

The present study results confirmed the hypothesis. The visual acuity, MTF cut-off, SR, OSI and subjective symptoms were better at 2 and 4 h after surgery, after the administration of 0.1% FML every half hour within two hours after SMILE, when compared to the administration of 0.1% FML every hour within two hours after SMILE. Furthermore, the more intensive regimen achieved the rapider restoration of visual and optical quality after SMILE. However, no difference was observed at 24 h and one week after SMILE. In the present study, the evaluated clinical outcomes were visual acuity, MTF cut-off, SR, OSI and subjective symptoms. Among them, MTF cut-off, SR and OSI were the objective indicators that reflected the opacity and its source in the optical media of the eyes. The SR was 0.15, and the MTF cut-off was ≥ 30 cpd in adults with normal vision, with a higher value representing better visual quality. Furthermore, the OSI was < 2 in adults with normal vision, with a higher value representing an opaque optical media and worse visual quality [8]. To the best of our knowledge, the use of such an intensive regimen in the very early phase after SMILE has not been reported in previous studies.

From the present study results, it was observed that the intensive topical steroid regimen accelerated the restoration of visual quality after SMILE and that the patients in group C were satisfied with the outcomes. According to the clinical experience of the investigators, these results are comparable to the early phase outcomes after FS-LASIK and are even better in some cases. As a limitation of the present study, the comparison between SMILE and FS-LASIK using an intensive steroid regimen was not performed. Therefore, more cases and studies are needed to further investigate this in future.

In conclusion, to the best of our knowledge, the present study was the first to involve an intensive topical steroid regimen in the very early phase after SMILE. The present study demonstrated that the administration of 0.1% FML eye drops every half hour within two hours after SMILE can accelerate the restoration of visual and optical quality. The improvement in the outcomes was reflected not only on the CDVA and subjective symptoms, but also on some of the objective optical indicators, such as the MTF cut-off, SR and OSI. The present study results confirm that the administration of 0.1% FML can improve the treatment regimen after SMILE and that this is favorable for the promotion of SMILE in clinical practice.

## Data Availability

The authors confirm that the data that support the findings of the study are available within the article.
